# Sex differences of post-Covid patients undergoing outpatient pulmonary rehabilitation

**DOI:** 10.1186/s13293-024-00609-z

**Published:** 2024-04-21

**Authors:** Alexander Kautzky, Stephan Nopp, Dietlinde Gattinger, Milos Petrovic, Martin Antlinger, Dustin Schomacker, Alexandra Kautzky-Willer, Ralf Harun Zwick

**Affiliations:** 1 Clinical Division of Social Psychiatry, Department for Psychiatry and Psychotherapy, Medical University if Vienna, Vienna, Austria; 2 Division of Insurance Medicine, Department of Clinical Neuroscience, Karolinska Institute, Stockholm, Sweden; 3 Clinical Division of Haematology and Haemostaseology, Department of Medicine I, Medical University of Vienna, Vienna, Austria; 4 Outpatient Pulmonary Rehabilitation, Therme Wien Med, Vienna, Austria; 5 Ludwig Boltzmann Institute for Rehabilitation Research, Vienna, Austria; 6 Clinical Division of Endocrinology and Metabolism, Department of Medicine III, Medical University of Vienna, Vienna, Austria

## Abstract

**Background:**

Following years of pandemic severe acute respiratory syndrome coronavirus 2 infections labelled Covid-19, long lasting impairment summarized as post-Covid syndrome (PCS) challenges worldwide healthcare. Patients benefit from rehabilitation programs, but sex specific aspects of improvement remain little understood. The aim of the study was to assess whether women and men differ in response to outpatient pulmonary rehabilitation for PCS.

**Methods:**

263 (54.4% female) patients partaking in outpatient pulmonary rehabilitation (OPR) due to PCS between March 2020 and July 2022 were included in a prospective observational cohort study. Outcomes were assessed at baseline and before discharge from OPR and included six-minute walking distance (6MWD), 1-second forced expiratory volume (FEV1), diffusion capacity for carbon monoxide, maximal inspiratory pressure (MIP), dyspnea (medical research council scale), and post-Covid functional status scale (PCFS). Sexspecific changes in outcomes following OPR were assessed by linear mixed model and presented as mean differences (MD) with 95% confidence intervals. Linear regression was applied to test whether 6MWD correlates with PCFS and the minimal clinically important difference (MCID) in 6MWD regarding an improvement of at least one point in PCFS was computed with logistic regression.

**Results:**

Significant improvement throughout OPR was observed for all outcomes (all *p* < 0.0001). Despite less severe Covid-19 infections, PCFS scores remained higher in females after OPR (*p* = 0.004) and only 19.4% of women compared to 38.5% of men achieved remission of functional impairment. At baseline as well as after OPR, females showed higher symptom load compared to men in dyspnea (*p* = 0.0027) and scored lower in FEV1 (*p* = 0.009) and MIP (*p* = 0.0006) assessment. Performance in 6MWD was comparable between men and women. An increase of 35 m in 6MWD was computed as minimal clinically important difference to improve functional impairment.

**Conclusion:**

Both subjective symptoms such as fatigue and dyspnea and objective impairment in performance in pulmonary function were more frequently observed among women. Despite improvement throughout OPR in both women and men, the sex-gap in symptom load could not be closed as women less often achieved remission from functional impairment due to PCS. Intensified treatment of these symptoms should be considered in women undergoing rehabilitation for PCS.

**Supplementary Information:**

The online version contains supplementary material available at 10.1186/s13293-024-00609-z.

## Abstract (plain language summary)

While female sex is protective during the acute infection of Covid-19, women are at increased risk of developing post-Covid syndrome (PCS) even after only mild Covid-19 infections. Severity and frequency of symptoms such as fatigue and shortness of breath are known to be higher in women compared to men. Many different rehabilitation protocols are used for PCS, but a knowledge gap regarding sex related differences in rehabilitation success remains.

Both female and male patients with PCS undergoing outpatient pulmonary rehabilitation improved in the maximum walking distance achieved within 6 min and selfrated impairment in everyday living. Although women less frequently required inpatient treatment for acute Covid-19 infection, female patients with PCS showed higher impairment in everyday living, lower capacity of physical exercise and more frequent shortness of breath, fatigue and breathing muscle weakness. Only 19.4% of women compared to 38.5% of men achieved complete remission of impairment in everyday living. Our results show that women treated for PCS retain greater symptom burden and are at risk of unsuccessful rehabilitation, calling for more targeted treatment in female patients after Covid-19 infection.

**Supplementary Information:**

The online version contains supplementary material available at 10.1186/s13293-024-00609-z.

## Highlights

Six weeks of outpatient pulmonary rehabilitation successfully improved 6-minute walking distance, pulmonary function and Covid-19 related functional limitations in daily living.

Women achieved remission of functional limitations less often than men (19.4% vs. 38.5%).

Women reported more severe dyspnea and showed greater impairment of maximal inspiratory pressure and forced expiratory volume compared to men.

**Supplementary Information:**

The online version contains supplementary material available at 10.1186/s13293-024-00609-z.

## Introduction

The ongoing Covid-19 pandemic has led to nearly 800 million people being infected with the novel severe acute respiratory syndrome coronavirus 2 (SARS CoV-2) labelled Covid-19 (https://covid19.who.int/, as of March 17th, 2024). Many patients suffer from symptoms impeding daily living longer than three months after recovery from acute infection and the term post-Covid syndrome (PCS) was established to describe this post-viral condition [1]. Recent population-based studies [2] and pooled estimates from available research [3] suggested that at least 6.5% but up to 28% of patients with Covid-19 are facing PCS, translating to 40 to 150 million cases worldwide. A recent study accounting for symptoms that were already present before Covid-19 while controlling for similar symptoms reported by patients without Covid-19 confirmed that one in eight patients with Covid-19 suffer from PCS [4]. Fatigue, dyspnea, cognitive impairment and mood or anxiety symptoms were reported by about one-third to one-half of PCS patients [2–4]. While clinical presentation resembles post-viral syndromes that frequently followed previous coronavirus outbreaks, i.e., SARS and Middle East respiratory syndrome [5], the scale of affected people is unprecedented. Early longitudinal studies reported a mean duration of PCS between 4 and 9 months. However, in approximately 15% of PCS cases, symptoms persisted for at least one year after testing positive for Covid-19 [3].

Sex differences impact both Covid-19 and PCS [6]. Mainly due to biological factors such as higher angiotensin convertible enzyme 2 (ACE2) mediated by sex hormones but also due to more frequent preexisting cardiovascular comorbidities men have higher rates of hospitalization and mortality in the acute infection [7], while women present more frequently with PCS and report more often core symptoms such as of dyspnea and fatigue [8]. As a result, risk factors for acute Covid-19 severity such as older age and cardiovascular comorbidities have not proven useful for assessment of PCS risk. A meta-analysis confirmed female sex among the most impactful risk factors for PCS, that more frequently develops from mild Covid-19 and at younger ages in women compared to men [9]. Next to biological differences such as X-chromosome linked immunoreactivity and protective effects of sex hormones regarding initial symptom severity, gender variables may account for higher symptom persistence in women [10]. While sex differences in symptom prevalence are well-established [4], implications for treatment of PCS and functional outcomes are scarce. The need for standardized and early interventions for PCS patients is clearly recognized. However, an abundance of rehabilitation protocols are currently deployed [11]. Here, we follow up and expand in terms of sex differences on recently reported improvement in pulmonary symptoms, exercise capacity and functional outcomes after six weeks of outpatient pulmonary rehabilitation (OPR) targeted at PCS patients [12].

## Methods

### Sample

The sample consists of all patients treated for PCS following a PCR-positive Covid-19 infection between March 2020 and July 2022 at the rehabilitation center ThermeWienMed (https://www.thermewienmed.at). As recently described in detail [12], all patients received six weeks of OPR following respective Austrian guidelines [13]. In short, patients completed a total of 60 rehabilitation sessions (á 50 min) split over six weeks that included a net worth of 38 h of physical exercise including endurance, strength and inspiratory muscle training in addition to diagnostic appointments, and clinical-psychological and nutritional counseling.

### Baseline characteristics

At admission, next to age, sex and body mass index (BMI), presence or absence of diabetes mellitus (DM) type 1 and type 2, obesity, hyperlipidemia, arterial hypertension, diastolic dysfunction, coronary artery disease (CAD), hyperuricemia, asthma and depression (ICD-10: F32 or F33) were assessed. Severity of Covid-19 was coded mild or moderate if no inpatient treatment was required, severe if patients were admitted to hospital, and critical if patients needed intensive care. PCS symptoms were grouped into neurocognitive, musculoskeletal, gastrointestinal, cardiac and hematological symptoms, dyspnea, fatigue, autonomous dysregulation (assessed by Schellong test), lung residuals after Covid-19 (assessed by lung imaging), breathing muscle weakness (maximal inspiratory pressure (MIP) scoring below 60 and 70 mBar respectively for women and men) and diffusion impairment (assessed by diffusion capacity for carbon monoxide (DLCO) below 80% of predicted values).

### Outcome variables

Expert-measured outcomes included the six-minute walking distance (6MWD) [14], 1-second forced expiratory volume (FEV1), MIP and DLCO [15]. Patient reported outcome variables included the post-Covid functional status scale (PCFS) [16], ranging from 0 (no limitations) to 3 (unable to perform usual activities) in this outpatient sample. The PCFS is the currently most established patient-rated scale for functional impairment in PCS and has been validated as useful tool for measuring PCS-related reduced quality of life. It was specifically recommended for evaluation of rehabilitation [17, 18]. Further, the modified medical research council scale (mMRC) was used for dyspnea assessment. Outcomes were assessed both as absolute values and percentages of age- and sex-adjusted reference values (%^pred^). Further, percentages of patients scoring below 80% of predicted reference values are reported for each outcome variable.

### Statistics

Baseline characteristics were described by means and standard deviations (SD) for metric parameters and by counts and frequencies for factorial variables and tested for significance respectively by t- and chisquare tests. For PCS symptoms and comorbidities odds ratios (OR) and 95% confidence intervals (CI) were computed.

Longitudinal changes are presented descriptively both as raw values, such as 6MWD in meters, and in %^pred^. Mean differences (MD) are reported with 95% CI. Linear mixed effects models were computed as provided by the R package “lmer”. For each outcome variable, models were built with sex and severity of Covid-19 (dichotomized to inpatient vs. outpatient treatment) as between-subject variables and time-point (admission and discharge) as within- subject variable. Three-way interactions were computed, and patient identifier was included as random effect. In presence of significant interactions with sex, post-hoc linear mixed models were computed respectively in female and male patients with main effects of time-point and Covid-19 severity.

To assess the association between patient-reported (PCFS) and expert-measured (6MWD) primary outcomes, a generalized linear model was computed with change in PCFS in points as outcome variable and change in 6MWD in meters as predictor, adjusted for Covid-19 severity and sex. Post-hoc, Spearman correlations between change in PCFS and 6MWD were computed, stratified by significant covariates. The minimal clinically important difference (MCID**)** regarding 6MWD change in meters as predictor of successful reduction in PCFS by at least 1 point was assessed by the anchor method and with receiver operating characteristic curves built by univariate logistic regression.

Considering that post-Covid syndrome is a new phenomenon with high clinical urgency, all analyses were regarded as exploratory and a p-threshold of 0.05 was accepted for significance.

## Results

A total of 263 patients (142 female, 54.4%) treated for respiratory symptoms or functional limitations after confirmed Covid-19 infection in the OPR center between March 2020 and July 2022 were included for analysis and are detailed in Table 1. The average time between positive testing for Covid-19 and admission to OPR was 6.5 (± 4.3) and 5.6 (± 3.6) months respectively for female and male patients (*p* > 0.05). Women were on average five years younger (45.0 ± 12.4 vs. 50.2 ± 12.6 years, t = -4.8, *p* < 0.0001) and less likely to have been hospitalized for treatment of Covid-19 (14.1% vs. 42.1%, x^2^ = 24.7, *p* < 0.0001).

**Table 1:** Characteristics of the study sample stratified by sex and reported respectively for time of admission and discharge from outpatient rehabilitation. Differences between women and men at baseline were tested by t-test (metric variables) and chi-square test (factorial variables) and significance is indicated by * for *p* < 0.05, ** for *p* < 0.005 and *** for *p* < 0.0005

	Female (*n* = 142)		Male (*n* = 121)		Overall (*n* = 263)	
	Admission	Discharge	Admission	Discharge	Admission	Discharge
**Sample Characteristics**						
Age ***						
Mean (SD), years	45.0 (12.4)		50.2 (12.6)		47.3 (12.8)	
Covid-19 ***						
Outpatient, Mild	107 (75.4%)		56 (46.3%)		163 (62.0%)	
Outpatient, Moderate	15 (10.6%)		14 (11.6%)		29 (11.0%)	
Inpatient	14 (9.9%)		34 (28.1%)		48 (18.3%)	
Intensive Care	6 (4.2%)		17 (14.0%)		23 (8.7%)	
**Time to Outpatient Pulmonary Rehabilitation**						
Mean (SD), months	6.47 (4.32)		5.65 (3.57)		6.07 (3.99)	
Missing	34 (23.9%)		19 (15.7%)		53 (20.2%)	
**Body Mass Index**						
Mean (SD), kg/m^2^	26.4 (5.97)		27.5 (4.81)		26.9 (5.48)	
Overweight	40 (28.6%)		52 (43.0%)		92 (35.2%)	
Obesity	31 (22.1%)		21 (17.4%)		52 (19.9%)	
**Clinician-Rated Outcomes**						
6-Minute Walking Distance ***						
Mean (SD), meters	523.1 (84.3)	581.5 (95.9)	583.7 (103.0)	643.0 (102.0)	551.2 (98.1)	610.4 (103.0)
Below 80% Predicted	34 (24.6%)	12 (10.2%)	24 (19.8%)	4 (3.81%)	58 (22.4%)	16 (7.17%)
Missing	4 (2.8%)	24 (16.9%)	0	16 (13.2%)	4 (1.5%)	40 (15.2%)
Maximal Inspiratory Pressure ***						
Mean (SD), mBar	70.7 (23.6)	96.4 (26.0)	104.0 (28.6)	127.0 (28.5)	86.0 (30.8)	111.0 (31.3)
Below 80% Predicted	49 (35.3%)	13 (9.2%)	9 (7.4%)	1 (0.8%)	58 (22.1%)	14 (5.3%)
Missing	3 (2.1%)	20 (14.1%)	1 (0.8%)	14 (11.6%)	4 (1.5%)	34 (12.9%)
1-Second Forced Expiratory Volume ***						
Mean (SD), liters	2.58 (0.62	2.68 (0.59)	3.36 (0.92)	3.60 (0.93)	2.94 (0.86)	3.11 (0.89)
Below 80% Predicted	52 (38.5%)	37 (29.4%)	29 (24.6%)	14 (12.7%)	81 (32.0%)	51 (21.6%)
Missing	7 (4.9%)	16 (11.3%)	3 (2.5%)	11 (9.1%)	10 (3.8%)	27 (10.3%)
**DLCO**						
Mean (SD), %-predicted	86.1 (17.4)	87.9 (20.0)	83.3 (17.8)	88.6 (18.0)	84.8 (17.6)	88.2 (19.1)
Below 80% Predicted	39 (32.8%)	34 (30.4%)	39 (37.9%)	26 (27.7%)	78 (35.1%)	60 (29.1%)
Missing	23 (16.2%)	30 (21.1%)	18 (14.9%)	27 (22.3%)	41 (15.6%)	57 (21.7%)
**Patient-Rated Outcomes**						
Post-Covid Functional Status Scale **						
Mean (SD), points	2.27 (0.80)	1.38 (0.94)	1.97 (0.93)	1.03 (0.98)	2.13 (0.87)	1.22 (0.97)
Moderate/Severe (≥ 2)	115 (85.2%)	58 (45.0%)	88 (77.2%)	37 (33.9%)	203 (81.5%)	95 (39.9%)
Missing	7 (4.9%)	13 (9.2%)	7 (5.8%)	12 (9.9%)	14 (5.3%)	25 (9.5%)
Dyspnea (mMRC) **						
None	38 (28.4%)	67 (53.2%)	49 (40.8%)	78 (67.8%)	87 (34.3%)	145 (60.2%)
I, Exertion	46 (34.3%)	48 (38.1%)	45 (37.5%)	32 (27.8%)	91 (35.8%)	80 (33.2%)
II - III, Walking	50 (37.3%)	11 (8.73%)	26 (21.7%)	5 (4.35%)	76 (29.9%)	16 (6.64%)
Missing	8 (5.6%)	16 (11.3%)	1 (0.8%)	6 (5.0%)	9 (3.4%)	22 (8.4%)

Abbreviations: SD = standard deviation, DLCO = diffusion capacity for carbon monoxide, mMRC = modified medical research council

Women presented more often with dyspnea (mMRC score ≥ 2 in 37.3% vs. 21.7%, x^2^ = 7.9, *p* = 0.005), fatigue (81.0% vs. 64.5%, x^2^ = 8.30 *p* = 0.004), autonomous dysregulation (23.7% vs. 11.6%, x^2^ = 4.0, *p* = 0.047) and respiratory muscle weakness (35.3% vs. 7.4%, x^2^ = 27.0, *p* < 0.0001), while lung residuals were more often observed in men (31.4% vs. 20.4%, x^2^ = 3.6, *p* = 0.047). This pattern was observed both among patients hospitalized during acute Covid-19 infection and milder cases, detailed in Supplementary Table 1.

**Fig. 1:**
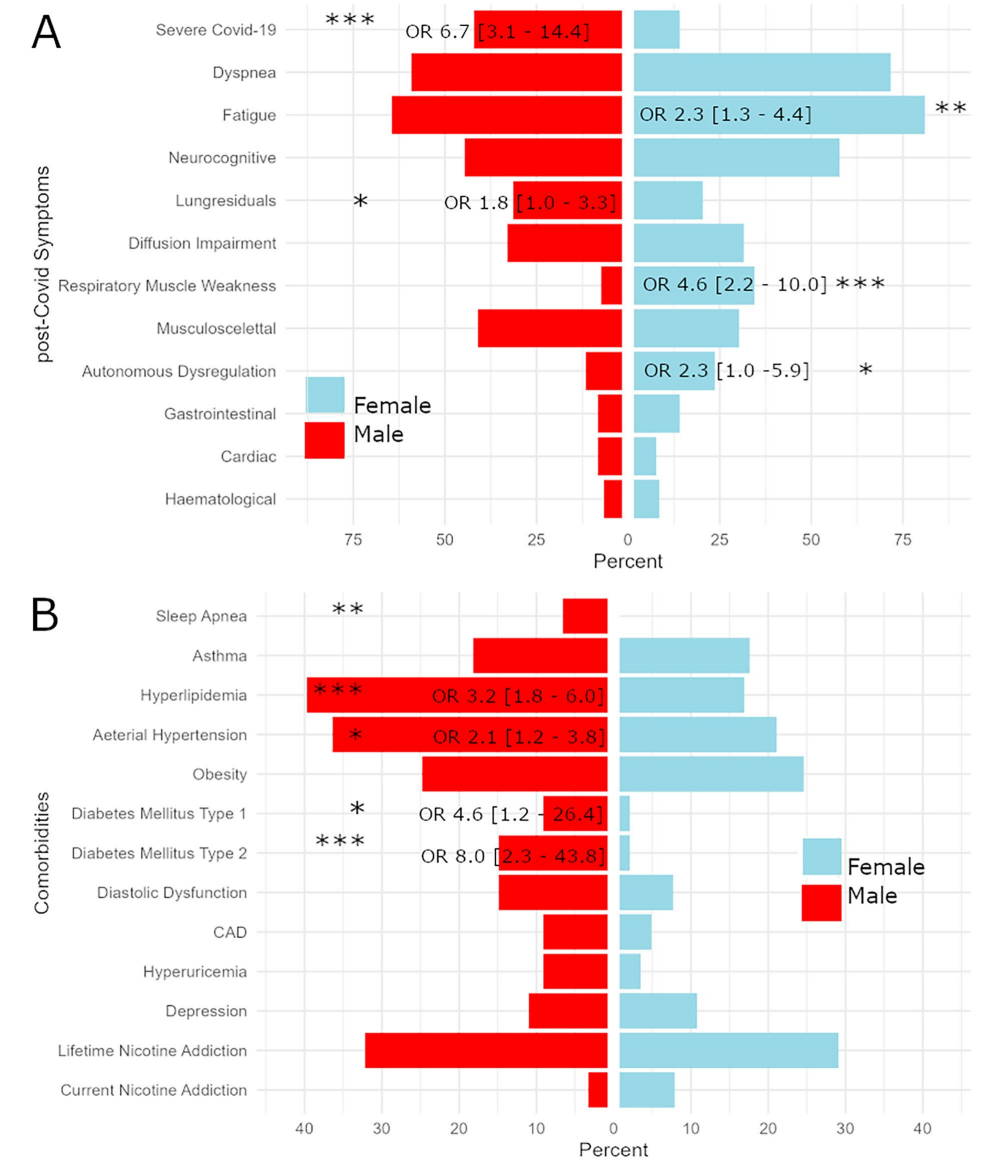
Bar chart opposing frequencies of post-Covid symptoms (**panel A**) and comorbidities (**panel B**) observed in female patients to that in males. Whenever significant differences in males and females were computed, odds ratios (OR) and 95% confidence intervals (CI) are presented. Significance levels are indicated by * corresponding to *p* < 0.05, ** to *p* < 0.005 and *** to *p* < 0.0005. Abbreviations: CAD = coronary artery disease

Regarding comorbidities, men were more often affected by DM type 1 (9.1% vs. 2.1%, x^2^ = 5.0, *p* = 0.025) and DM type 2 (14.9% vs. 2.1%, x^2^ = 12.8, *p* = 0.0003), arterial hypertension (36.4% vs. 21.1%, x^2^ = 6.8, *p* = 0.009), hyperlipidemia (39.7% vs. 16.9%, x^2^ = 15.9, *p* = 0.0001) and obstructive sleep apnea (6.6% vs. 0%, Fisher test: *p* = 0.018), while BMI, presence of obesity and major depressive disorder did not differ between men and women. Please also refer to Fig. 1 for sex-related OR with CI for baseline characteristics.

### 6MWD

**Fig. 2:**
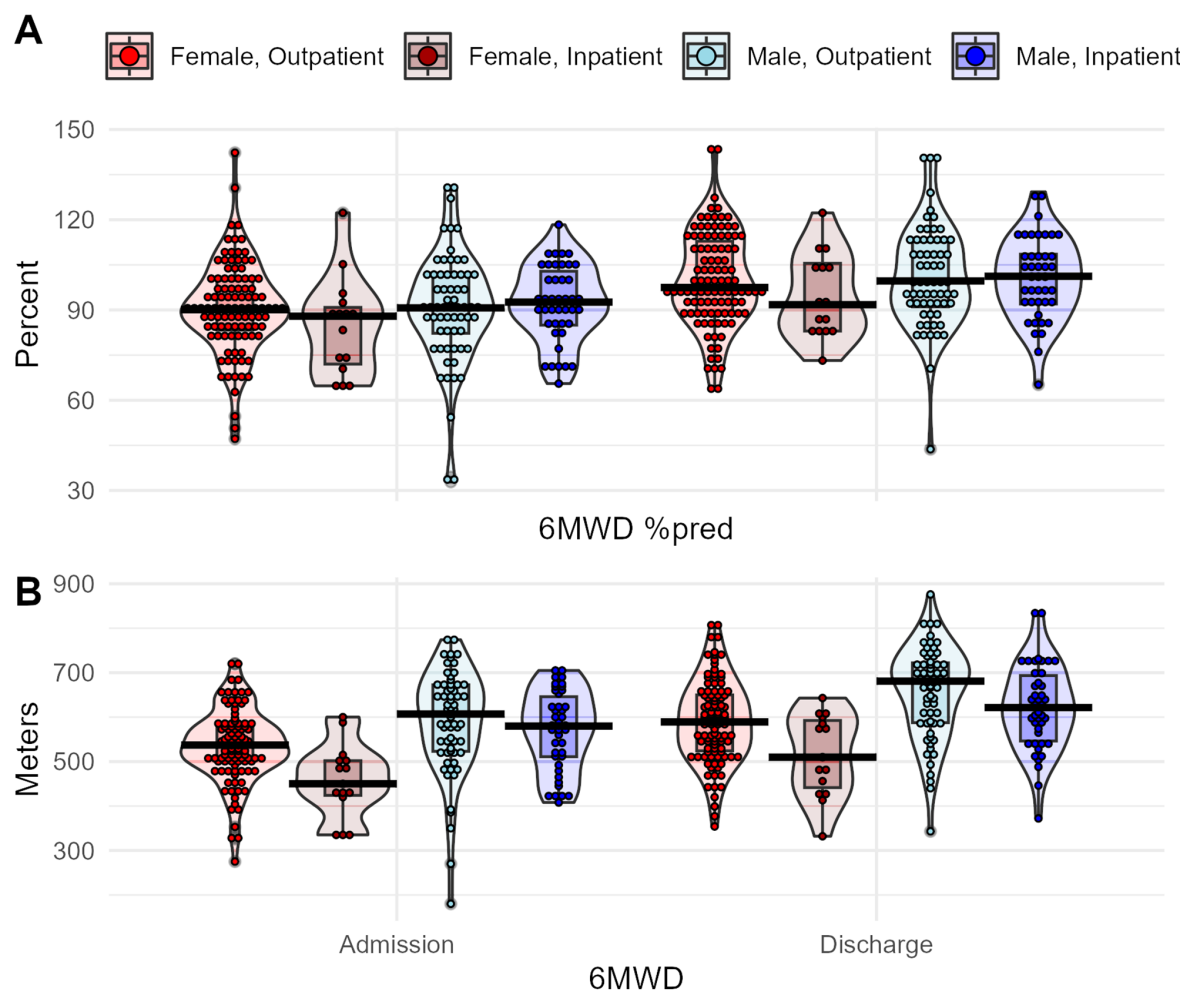
Boxplot diagrams of physical performance assessed by 6-minute walking distance (6MWD). Colors indicate sample stratification by sex and severity of Covid-19. Panel A shows the ratio between achieved distance and predicted values based on age- and sex-adjusted reference equations, panel B raw values in meters walked

On average, at admission women walked 525.2 m (± 85.5) and men walked 581.9 m (± 106.25) of 6MWD. Before OPR, women scored 8.5%-points (CI 0.7–16.3) lower in 6MWD^%pred^, and 24.6% of women compared to 19.8% of men ranked below 80% of 6MWD^%pred^. Patients improved over time (F^1, 218^ = 238.6, *p* < 0.0001), while no main or interaction effects of sex or Covid-19 severity were observed. At discharge from OPR, the MD in 6MWD^%pred^ between females and males was 5.6%-points (CI 2.5–13.8) and 10.2% of women compared to 3.8% of men still scored below 80% of their predicted reference values. Please also refer to Fig. 2 for violin plots depicting performance in 6MWD. A complete list of mixed model results is provided in Table 2.

**Table 2:** Mixed model analysis of variance (ANOVA) results for all outcomes. All models included main effects of time-point (admission and discharge from outpatient pulmonary rehabilitation), sex, severiy of Covid-19 (outpatient vs. inpatien), as well as their interactions. Only significant interactions are listed, which were followed up by stratification of the sample by sex. For stratified cohorts, only sognificant effects are listed

Outcome	Stratification	Degrees of Freedom	*F*	*p*
**6-Minute Walking Distance, % predicted**				
Sex				n.s.
Time-Point		1, 218	238.6	< 0.0001
Covid-19				n.s.
**1-Second Forced Expiratory Volume, % predicted**				
Sex				n.s.
Time-Point		1, 227	24.3	< 0.0001
Covid-19		1, 228	4.6	0.032
Sex * Time-Point		1, 227	3.9	0.051
Sex * Covid-19		1, 227	6.9	0.009
	**Males**			
	Time-Point	1, 106	16.2	0.001
	Covid-19	1, 106	5.6	0.019
	**Females**			
	Time-Point	1, 121	8.2	0.005
**Maximal Inspiratory Pressure**				
Sex		1, 225	12.1	0.0006
Time-Point		1, 225	340.6	< 0.0001
Covid-19				n.s.
Sex * Time-Point		1, 225	5.9	0.0158
	**Males**			
	Time-Point	1, 105	136.7	< 0.0001
	**Females**			
	Time-Point	1, 120	204	< 0.0001
**Diffusion Capacity of Carbon Monoxide, % predicted**				
Sex				n.s.
Time-Point		1, 181	16.2	0.0001
Covid-19		1, 181	22.3	< 0.0001
**Dyspnea (Medical Research Council Scale)**				
Sex		1, 237	9.2	0.0027
Time-Point		1, 237	160.1	< 0.0001
Covid-19		1, 237	10.4	0.0014
Sex * Covid-19		1, 237	2.9	0.0510
	**Males**			
	Time Point	1, 113	65.2	< 0.0001
	**Females**			
	Time Point	1, 124	96.7	< 0.0001
	Covid-19	1, 132	11.0	0.0012
**post-Covid Functional Status Scale**				
Sex		1, 234	8.3	0.0043
Time-Point		1, 234	295.8	< 0.0001
Covid-19				n.s.

### Pulmonary outcomes

Interactions effects between sex and respectively time-point (F^1, 227^ = 3.9, *p* = 0.051) and Covid-19 severity (F^1, 227^ =6.9, *p* = 0.009) were computed for FEV1^%pred^. Despite the lack of a significant threeway interaction, sex differences in improvement were observed mostly in patients with severe Covid-19 infection that showed a MD between time-points of 124%points in men compared to 0.2%-points in women. Regarding DLCO, improvement over time (F^1, 181^ = 16.2, *p* = 0.0001) but no main or interaction effects of sex were observed.

A significant interaction between sex and time-point was computed for MIP^%pred^ (F^1, 225^ = 5.9, *p* = 0.016). MIP^%pred^ was decreased in women compared to men by a MD of 16.5%-points (CI 9.3–23.6) at admission. Despite stronger relative improvement in females shown by average change between time-points of 31.5%-points (CI 24.2–38.8) compared to 23.9%-points (CI 16.5–31.4) in men, women still scored lower MIP^%pred^ compared to men at discharge by a MD of 8.9%-points (CI 1.3–16.6). Inspiratory muscle weakness was present in 34.5% of women but only 7.4% of men at baseline. After rehabilitation, 9.2% of women and a single man still fulfilled criteria for inspiratory muscle weakness.

Women reported more dyspnea on the mMRC scale (F^1, 237^ = 9.2, *p* = 0.003) compared to men, both at admission (MD 0.3, CI 0.1–0.5) and after rehabilitation (MD 0.2, CI 0.04–0.4). Further, an interaction effect on mMRC score between Covid-19 infection severity and sex was found (F^1, 237^ = 2.9, *p* = 0.051). Only among women, severe Covid-19 was linked to higher mMRC scores (F^1, 132^ = 11.0, *p* = 0.001). Please also refer to Supplementary Figs. 3–5 for violin plots depicting pulmonary outcomes.

### PCFS scale

At admission to OPR, 85.2% of female and 77.2% of male patients reported a PCFS score ≥ 2 indicating clinically relevant functional limitations. An increase of at least one point in PCFS was achieved by 68.5% of female compared to 74.5% of male patients. Despite improvement in PCFS over time both in women and men (F^1, 234^ = 295.8, *p* < 0.0001), female patients showed more severe limitations in daily living compared to men (F^1, 234^ = 8.3, *p* = 0.004). At admission, 44.2% of women scored PCFS of 3 compared to 30.7% of men. At discharge, clinically relevant impairment indicated by PCFS score ≥ 2 was still reported by 45.0% women compared to 33.9% of men. Remission (PCFS of 0) was also achieved by fewer women (19.4%) than men (38.5%).

**Fig. 3:**
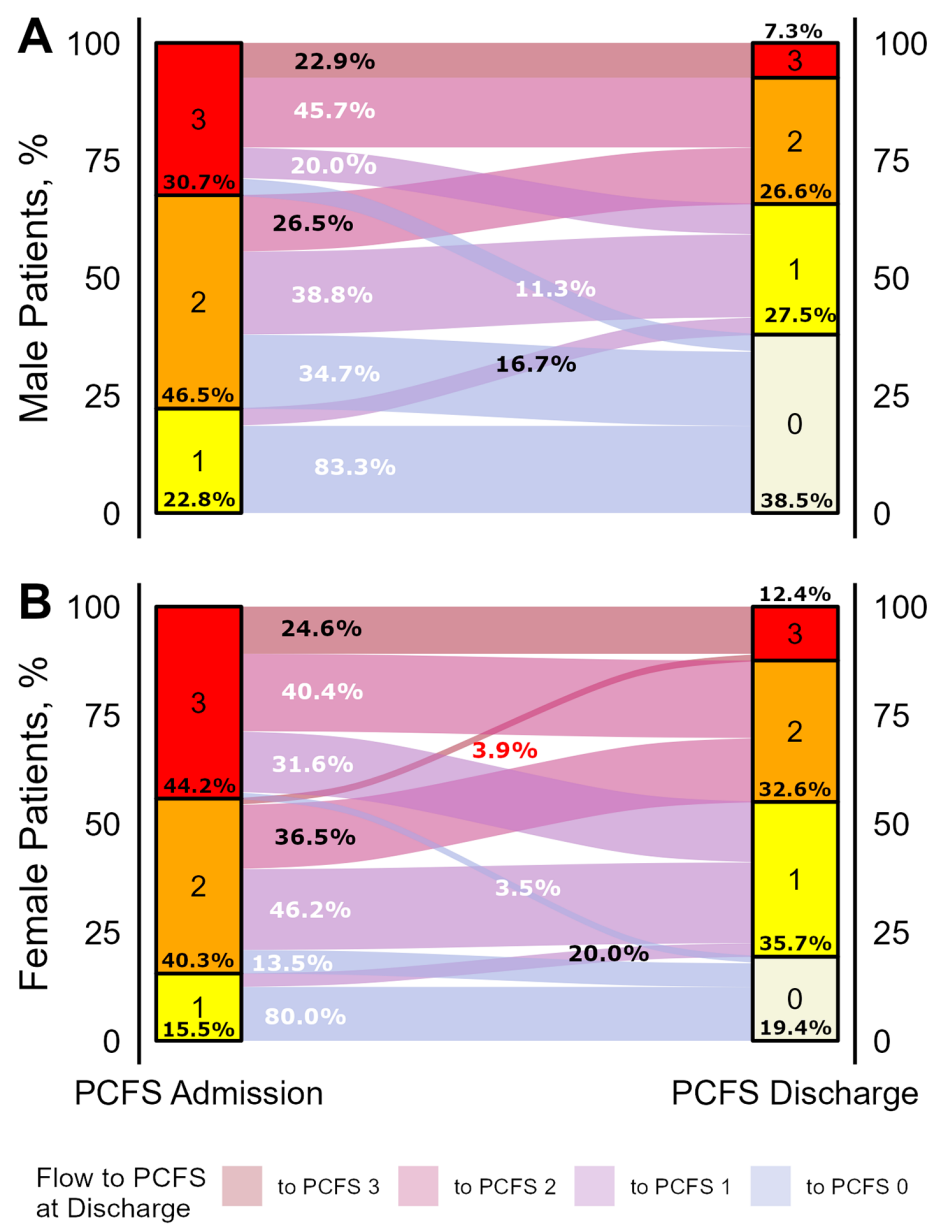
Flow diagram showing changes in post-Covid functional status (PCFS) scale between admission and discharge from pulmonary outpatient rehabilitation. Flow trajectories are sized according to the patient numbers following them and colors indicate the PCFS score at discharge. Numbers indicate percentages of patients flowing from each stratum of PCFS score at admission, respectively colored white for patients that achieve a drop of at least one point in PCSF score, black for patients with stagnant PSCFS score, and red for patients with worsened PCFS scores at discharge. Example given, 3.5% of female patients compared to 11.3% of male patients with a PCFS score of 3 at admission achieved full remission indicated by a PCSF score of 0

In summary, 60.1% of patients reported PCFS ≤ 1 after OPR and thus benefitted from rehabilitation. A quarter of the remaining 39.9% of patients in need of further rehabilitation can be considered non-responders to OPR as they still scored PCFS of 3 at discharge. Only two patients worsened in PCFS over the course of OPR, both of which were women. Please refer to Fig. 3 for an alluvial plot detailing changes in PCFS scores respectively for women and men.

Absolute change in PCFS was associated with the interaction between absolute change in 6MWD in meters and severity of Covid-19 (F = 5.4, *p* = 0.021). The association between changes in 6MWD and PCFS was only present in those treated as inpatients for severe Covid-19 infection (F = 10.3, *p* = 0.002). Changes in PCFS and 6MWD showed a moderate Spearman correlation in patients who had severe (*r* = 0.41, *p* = 0.003) but not in those with mild to moderate (*r* = 0.07, *p* > 0.05) Covid-19 infection (Supplementary Fig. 1). An improvement of 35 m in 6MWD was identified as MCID, corresponding to an increase of at least one point in PCFS, and was achieved by 61.5% of women and 63.8% of men. A sensitivity of 72.5% and specificity of 53.5% was achieved to classify patients with and without improvement of PCFS (Supplementary Fig. 2). In the subsample of patients with severe Covid-19 infection, the same cut-off of 35 m allowed better performance with a sensitivity 77.8% and specificity of 75%.

## Discussion

In consecutive patients with PCS undergoing six weeks of OPR, significant improvement was observed in exercise capacity and respiratory function. These results reinforce our previous report on the success of OPR in a preliminary sample of 64 patients [12], while emphasizing the importance of sex differences in PCS symptom presentation and outcome. Women showed worse functioning in daily activities measured by PCFS. Rehabilitation failed to bridge the gap separating them from men regarding PCFS scores, symptom burden of dyspnea, and objectified performance in MIP.

The rate of hospitalization during the acute Covid-19 infection was considerably lower among women (14.1%) compared to men (42.1%), which is in line with higher morbidity and mortality in men during acute Covid-19 infection demonstrated in prior studies [7]. These sex-gaps were attributed to biological differences in women and men such as higher ACE2 in men that is used by SARS-CoV-2 for cell entry [19]. Thereby, male sex hormones testosterone and dihydrotestosterone were suggested to upregulate ACE2, suppress immune responses and increase endothelial damage in Covid-19. Prolonged hospitalization and admission are known to bring along cardiorespiratory sequelae and a need for rehabilitation, suggesting higher need of OPR in men following Covid-19. However, in this cohort of patients with PCS undergoing OPR both physical and functional limitations were significantly higher in women despite having suffered predominantly mild acute infections of Covid-19. Our results agree with consistent observations that women develop Covid-19 more often than men following mild infections and that initial severity is of limited prognostic value for PCS [20]. Importantly, women did not only endorse more subjective symptoms such as fatigue and dyspnea as previously reported [20–22] but also showed higher rates of objective pulmonary impairment such as decreased inspiratory muscle strength throughout OPR.

The reasons for observed sex differences are less understood in PCS compared to acute Covid-19 infections. Fatigue is a central symptom of affective disorders such as depression as well as syndromes associated with exposure to viral infections known prior to Covid-19 such as myalgic-encephalomyelitis/chronic-fatigue-syndrome (ME/CFS) [23, 24]. A sex-gap with female overrepresentation is well-documented in both depression and ME/CFS and is putatively owed to both gender-related variables and biological factors. Conversely to acute virus infections that more severely affect men, women may be disadvantaged regarding post-viral syndromes such as ME/CFS and PCS by prolonged immune responses that lead to endothelial dysfunction [19]. Further, sex differences regarding responses of the hypothalamic-pituitary-adrenal (HPA) axis to acute and chronic stress are well-established and enhanced activity in women was suggested as contributing factor to higher rates of stress-related disorders such as depression [25]. Direct impairment of the pituitary gland and HPA dysregulation may occur during Covid-19 and cause typical symptoms of PCS such as fatigue in sex-dependent manner [26].

Furthermore, sex hormones and particularly low estrogen were previously associated with depression and ME/CFS and may also be relevant to PCS considering that symptoms such as fatigue and low mood are shared with menopause [27]. Interestingly, most pronounced sex differences in PCS symptom presentation were observed in patients below 50 years of age in a cohort followed-up after hospitalization due to Covid-19. Here, the average age of women was 45 years which suggest perimenopausal states in a relevant portion of female patients. Disruptions of the female menstrual cycle with transient disturbance of sexual hormones were observed inconsistently in PCS and may in part be responsible for symptoms predominantly seen in women with PCS [24].

Besides biological sex, gender roles typically assumed by women also contribute to clinical differences observed in PCS. Gender perspectives on Covid-19 were rarely considered despite early calls for implementation reflecting on the foreseeably disproportionate impact of the pandemic on female and male working- and social-life [28]. Particularly in non-hospitalized patients with Covid-19, female gender measured by a composite score was a stronger predictor for PCS than biological sex [20]. Living alone was a strong predictor for PCS in women but a protective factor in men, indicating interplay of socioeconomic and psychosocial factors. The role of gender in rehabilitation is still unknown and calls for further research.

Along these lines, psychiatric comorbidities preexisting Covid-19 infection more frequently in women than men due to gender and sex-related risk factors may explain differences in PCS presentation. While rates of depression documented prior to admission were comparable between women (10.6%) and men (10.7%) in this sample, higher rates of psychiatric comorbidities in women with PCS was recently reported by a large epidemiological sample [20]. Both in treatment of depression and rehabilitation, fatigue is known to be an unfavorable prognostic marker [29]. The higher rates of fatigue observed in women at baseline may therefore indicate a higher load of newly onset or aggravated neuropsychiatric symptoms that require specialized treatments.

Regarding functional limitations, the PCFS scale was designed to comprehensively rate impairment in daily activities in PCS patients and was applied in a broad spectrum of studies [16–18]. At admission, on average 6 months after Covid-19 infection, 44.2% of female compared to 30.7% of male patients reported severe impairment (PCFS of 3). As changes in PCFS were similar in women and men, higher impairment in women was still observed after completing rehabilitation. Stratification by baseline functional limitations revealed similar trajectories for men and women presenting with mild impairment (PCFS of 1), showing remission in 83% and 80% of cases. However, respectively with moderate (PCFS 2) and severe (PCFS 3) limitations at admission, 34.7% and 11.4% of men showed complete remission, compared with 13.5% and 3.5% of women. On the other hand, a stagnant PCFS score indicating resistance to rehabilitation was seen in 30.2% of women and 22.9% of men. In summary, more than a third of male patients (38.5%) achieved complete remission of functional impairment (PCFS of 0), compared to less than a fifth of female patients (18.9%).

Despite reporting more functional impairment, women did not underperform regarding 6MWD. Scores below 80% of 6MWD^%pred^, commonly used as threshold of norm values, were seen in 22.8% of patients at admission to rehabilitation. Roughly three-quarters of these clinically impaired patients successfully improved their walking distance to norm ranges until discharge. These numbers as well as absolute 6MWD are in line with findings in 83 Chinese patients followed up three and six months after inpatient treatment for severe Covid-19 in Wuhan [30], but diverge from reports of PCS patients with substantially lower 6MWD of 461 m [31], and cohorts with up to half of patients scoring 6MWD below 80% [32]. Females may thereby be at increased risk for scoring below the norm threshold [33]. On the other hand, an Italian study stratifying 75 PCS patients by Covid-19 symptom load reported mostly unimpaired 6MWD, even in the severe symptoms group [34]. Another study comparing PCS patients to Covid-19 negative patients matched by sex, age and cardiovascular profile also observed similar performance in 6MWD [35]. A comprehensive study on all confirmed cases with desaturated oxygen below 94% in Iceland drew a more distinct picture with lowered 6MWD observed only in patients treated in intensive care during Covid-19 [36]. This finding was supported by a study comparing intensive care to other hospitalized patients [37]. This distinction is also observed in the present study. Compared to outpatients and non-intensive-care inpatients, especially female (22.7% in non-intensive care vs. 66.6% in intensive care) but also male (18.2% vs. 29.4%) patients that had been admitted to intensive care units showed considerably higher rates of impaired 6MWD. Regardless of these considerations, rehabilitation programs targeting PCS were demonstrated to successfully raise 6MWD [12, 38–40]. Patients undergoing three weeks of cardiopulmonary rehabilitation in Poland improved on average 42.5 m, a comparable finding to the 55 and 61 m observed here respectively for women and men after six weeks [39]. However, the clinical importance of 6MWD as a marker for functional outcome in PCS can be questioned considering both that a significant portion of PCS patients show 6MWD within the normal ranges and that especially women often remain clearly impaired despite achieving an improvement of 35 m or more deemed clinically relevant. Change in 6MWD showed moderate correlation with change in PCFS score in patients with severe but poor correlation in patients with mild to moderate Covid-19 infection. Hence, we argue that 6MWD does not provide a complete picture of rehabilitation success and likely reflects subjective improvement only in patients that were hospitalized for treatment of their Covid-19 infection.

Pronounced sex differences were observed in respiratory muscle strength assessed by MIP. that are in line with previous reports of impairment in women with PCS [41]. Scores in MIP below the established ranges of 60 mbar for women and 70 mbar for men were observed in 35.4% of females but only 7.4% of males. Despite relatively stronger improvement in females, still 9.2% of women compared to a marginal 0.8% of men remained in the clinically relevant low range. Regarding FEV1, considerably worse scores in patients with severe Covid-19 were observed. Interestingly, male patients with severe Covid-19 successfully closed the gap in FEV1 separating them from those with mild Covid-19 during OPR, while female patients did not. Regarding subjective pulmonary symptoms, women reported higher mMRC scores throughout the observation period. Female sex was previously linked to pulmonary symptoms [30, 41]. Here, roughly 80% of patients that presented moderate to severe dyspnea at admission successfully improved over the course of OPR. However, only 47.2% of women compared to 64.5% of men showed complete remission of dyspnea, potentially due to higher rates of impaired inspiratory muscle strength in women. In synopsis, OPR was effective in improving pulmonary outcomes especially in patients with higher impairment, i.e., females regarding MIP and patients with severe Covid-19 regarding FEV1. Nevertheless, more severe residual impairment in women calls for targeted interventions.

Reflecting on these results, selection bias must be considered as the most important limitation. Considerable differences in baseline symptoms across rehabilitation services indicate ambiguity in patient allocation [40], although the pattern of sex-differences resembles well-replicated findings of PCS more often manifesting in women following mild infections [20]. Despite controlling for the severity of the acute Covid-19 infection, we cannot fully rule out that differences in OPR outcomes were driven by earlier phases of the infection. Further, only patients eligible for outpatient rehabilitation were included in this analysis and thus different patterns may be observed in inpatient rehabilitation. Some studies have suggested that sex-differences to be less pronounced in hospitalized and elderly patients potentially due a stronger role of cardiovascular comorbidities that cross out some of the sex- and gender-related effects demonstrated in PCS [20]. Consequently, we cannot generalize the findings presented here to other cohorts of PCS patients.

Furthermore, other follow-up studies at various time points after Covid-19 observed improvement of physical performance and to some extent of typical PCS symptoms as a function of time rather than rehabilitation [30]. While controlled trials are exceedingly rare, a study matching confirmed Covid-19 cases to patients without Covid-19 but similar other risk factors observed no differences in standard assessments such as 6MWD [35]. Hence, we cannot verify that the observed improvement was in fact caused by the rehabilitation program alone.

Further, reference equations that are commonly applied to resolve physiological sex- and age-related differences in performance were shown to lack congruency and are dependent on their data-context [42]. Finally, we cannot rule out false positive results due broad application of tests in an explorative manner.

### Perspectives and significance

OPR is demonstrated to be an effective and safe measure to facilitate subjective as well as objective recovery from PCS symptoms and impairment in daily activities. However, sex differences in PCS rehabilitation outcomes hold important implications for clinical practice. Women present more often with highly prevalent PCS symptoms fatigue and dyspnea and are more severely limited by these symptoms in daily living. Here we show that women and men show improvement during rehabilitation in all recorded outcomes, while underlining that more targeted protocols are called for to enable women to bridge the gap still separating them from more favorable outcomes observed in men at rehabilitation discharge. These may include earlier as well as modular interventions addressing sex differences in functional status and specific symptom presentations such as dyspnea and breathing muscle weakness.

### Electronic supplementary material

Below is the link to the electronic supplementary material.

Supplementary Material 1

## Data Availability

Data are available from the corresponding author on reasonable request.
