# Supplementary material for: Sex differences of post-Covid patients undergoing outpatient pulmonary rehabilitation
Source: Biol Sex Differ. 2024 Apr 21;15:36. doi: 10.1186/s13293-024-00609-z (PMC11034076; doi:10.1186/s13293-024-00609-z)
Supplement: Supplementary file 1 — Supplementary Material 1 [file 13293_2024_609_MOESM1_ESM.docx]

| **Outcome** | **Sex** | **Covid-19** | **Admission** | **SD** | **Discharge** | **SD** |
| --- | --- | --- | --- | --- | --- | --- |
| **6MWD,**  **meters** | Female | Outpatient | 535 | 81.5 | 591 | 93.3 |
|  |  | Inpatient | 456 | 82.3 | 515 | 91.8 |
|  | Male | Outpatient | 590 | 116 | 656 | 103 |
|  |  | Inpatient | 570 | 89.3 | 622 | 96.9 |
| **6MWD,**  **% predicted** | Female | Outpatient | 90.8 | 15.2 | 99.9 | 15.8 |
|  |  | Inpatient | 84.5 | 16.3 | 94.7 | 13.9 |
|  | Male | Outpatient | 90.7 | 18.6 | 101.0 | 16.9 |
|  |  | Inpatient | 92.2 | 12.7 | 100.5 | 13.7 |
| **Max. Inspiratory**  **Pressure, mBar** | Female | Outpatient | 71.5 | 23.8 | 96.3 | 25.9 |
|  |  | Inpatient | 77.4 | 22.8 | 97.1 | 27.3 |
|  | Male | Outpatient | 109.4 | 30.6 | 131.3 | 31.1 |
|  |  | Inpatient | 98.5 | 23.5 | 122.3 | 23.9 |
| **Max. Inspiratory**  **Pressure,**  **% predicted** | Female | Outpatient | 88.2 | 28.5 | 118.5 | 30.0 |
|  |  | Inpatient | 98.5 | 27.8 | 124.1 | 35.2 |
|  | Male | Outpatient | 109.0 | 30.8 | 130.5 | 29.6 |
|  |  | Inpatient | 101.3 | 25.7 | 125.7 | 25.3 |
| **FEV1, liters** | Female | Outpatient | 2.7 | 0.6 | 2.7 | 0.6 |
|  |  | Inpatient | 2.2 | 0.5 | 2.4 | 0.5 |
|  | Male | Outpatient | 3.6 | 0.8 | 3.8 | 1.0 |
|  |  | Inpatient | 3.0 | 0.8 | 3.4 | 0.8 |
| **FEV1,**  **% predicted** | Female | Outpatient | 86.7 | 17.0 | 89.5 | 17.2 |
|  |  | Inpatient | 80.9 | 15.7 | 86.1 | 16.5 |
|  | Male | Outpatient | 90.0 | 17.0 | 93.9 | 17.9 |
|  |  | Inpatient | 79.9 | 21.6 | 92.3 | 17.5 |
| **DLCO,**  **% predicted** | Female | Outpatient | 87.2 | 17.7 | 90.8 | 16.5 |
|  |  | Inpatient | 78.0 | 17.3 | 80.5 | 15.9 |
|  | Male | Outpatient | 90.0 | 14.8 | 92.1 | 14.3 |
|  |  | Inpatient | 73.9 | 18.6 | 79.2 | 16.6 |
| **mMRC** | Female | Outpatient | 1.0 | 0.9 | 0.5 | 0.7 |
|  |  | Inpatient | 1.6 | 0.8 | 1 | 0.6 |
|  | Male | Outpatient | 0.7 | 0.8 | 0.3 | 0.5 |
|  |  | Inpatient | 0.9 | 0.8 | 0.5 | 0.7 |

**Supplementary Table 1.** Mean values and standard deviations (SD) for all outcome variables, rspectively for time of admission and discharge from rehabilitation. The sample is furtehr startifed by severity of Covid-19, dichotomized to in- and outpatient treatment during acute infection. Abbreviations: 6MWD = 6-minute walking distance, FEV1  = 1-minute forced expiratory volume, mMRC = modified medical research council, STST = 1-minute sit-to-stand test, DLCO = diffusion capacity of carbon monoxide

**
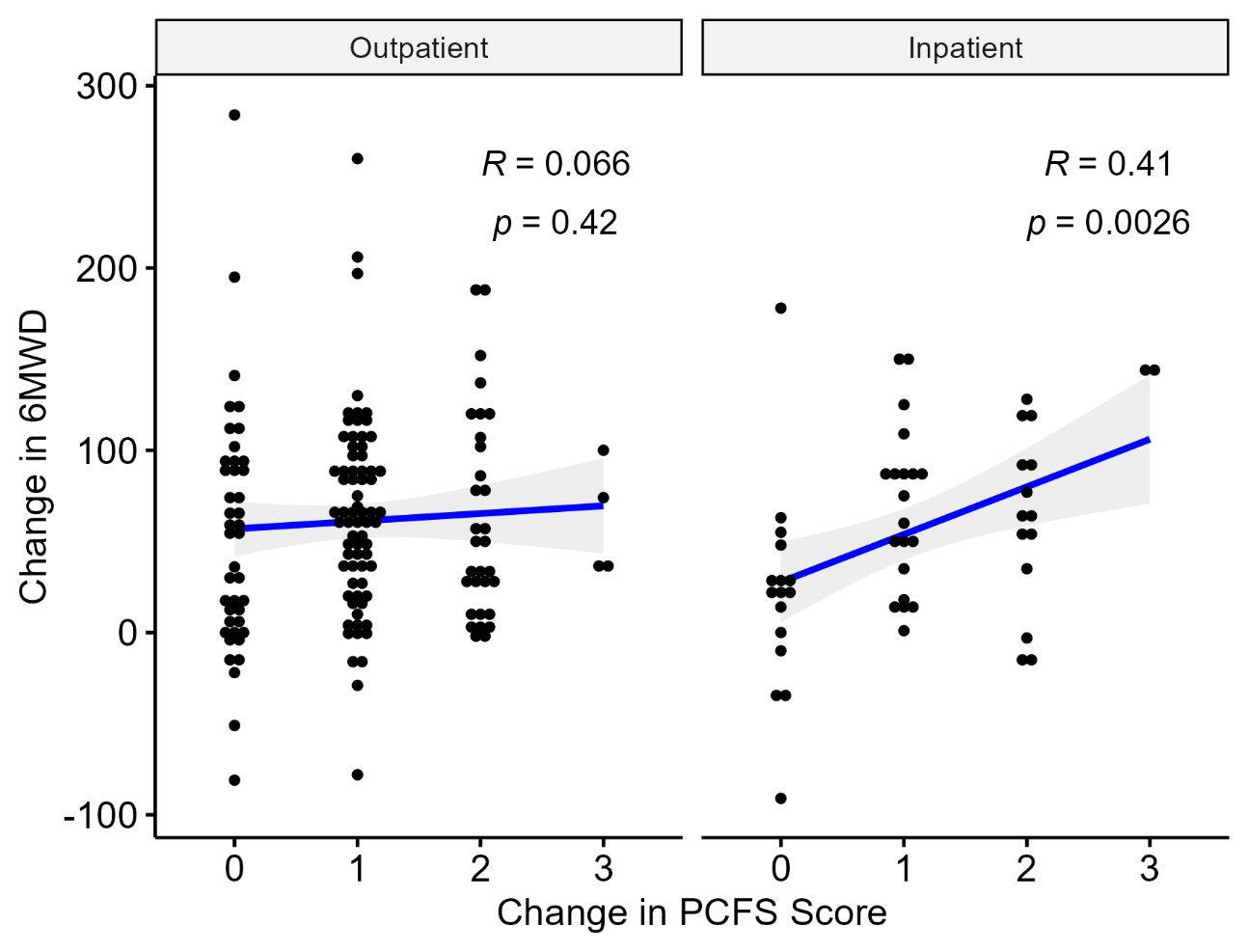
**

**Supplementary Figure 1.** Spearman correlation of change in PCFS score with change in 6MWD. Correlation coefficients (R) and p-values are reported. The left panel shows correlation for patients treated as outpatients during the Covid-19 of SARS-CoV2 infection, the right panel for inpatients.


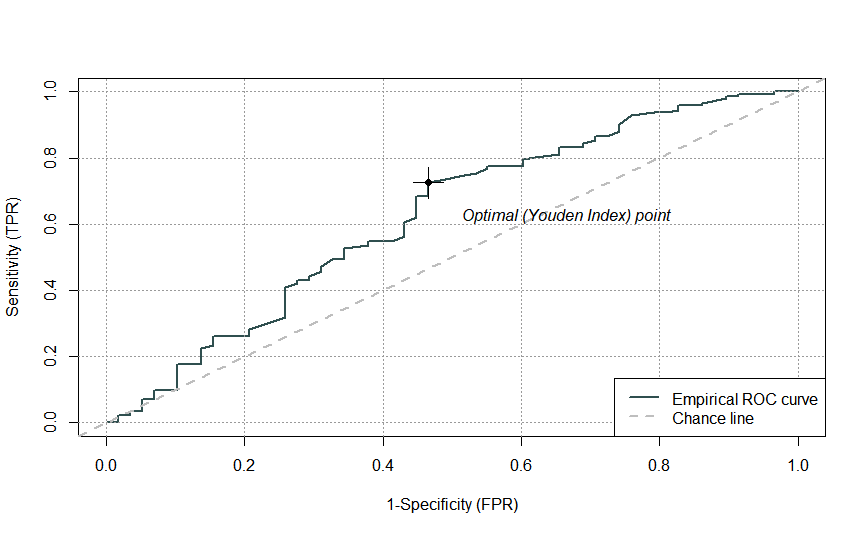


**Supplementary Figure 2**. Receiver operator curve showing the optimal cut-off in achieved improvement in 6-minute walking distance (6MWD) for predicting a change of at least one point in post-Covid functional status scale. The best tradeoff between true positive rate (TPR) and false positive rate (FPR) was computed at 35 meters improvement in 6MWD.


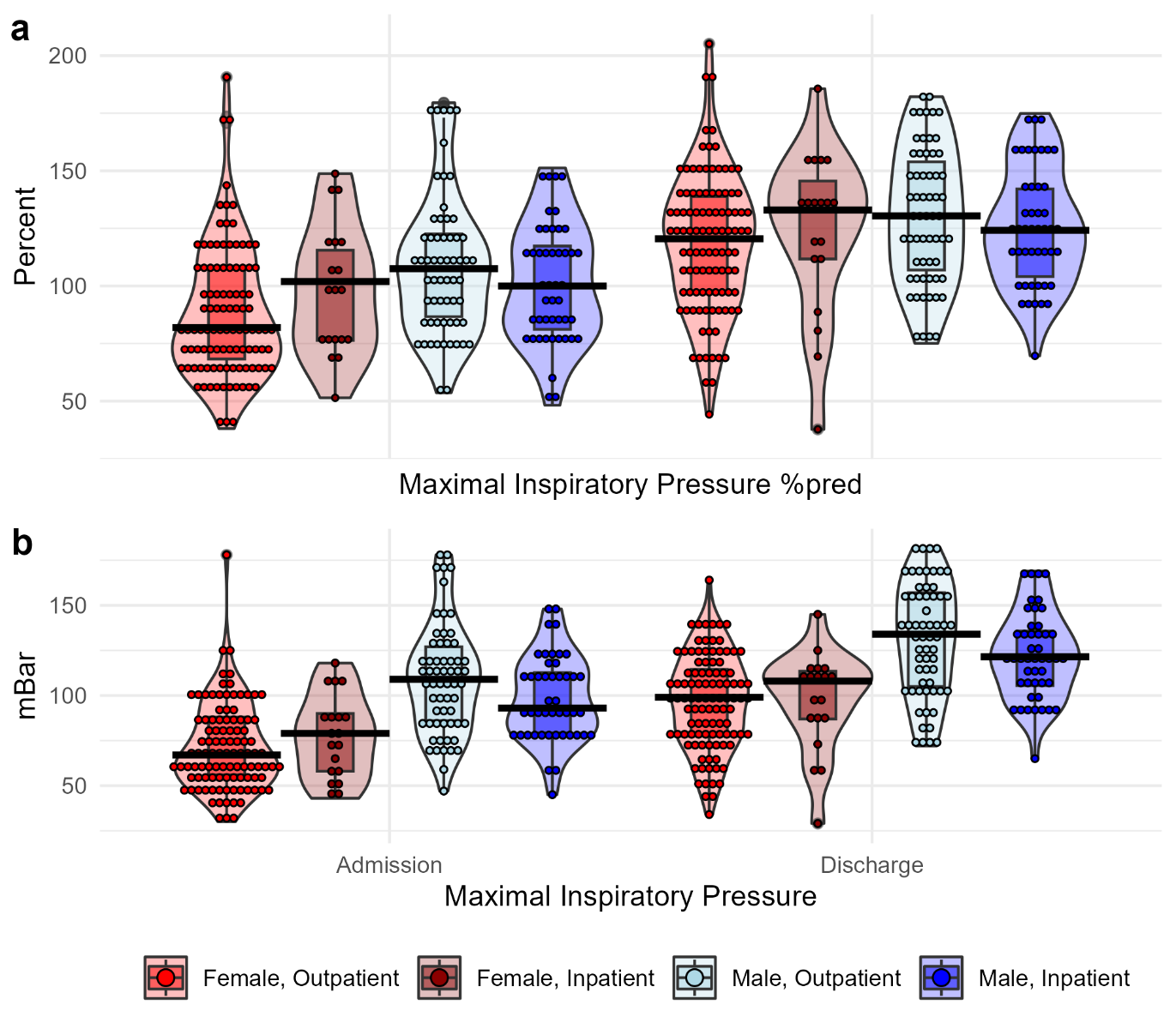


**Supplementary Figure 3**. Violin boxplot diagrams of maximal inspiratory pressure. Colors indicate sample stratification by sex and acute SARS-CoV2 infection severity. Panel a) shows the ratio between achieved mBar and predicted values based on age- and sex-adjusted reference equations, panel b) raw values in mBar.


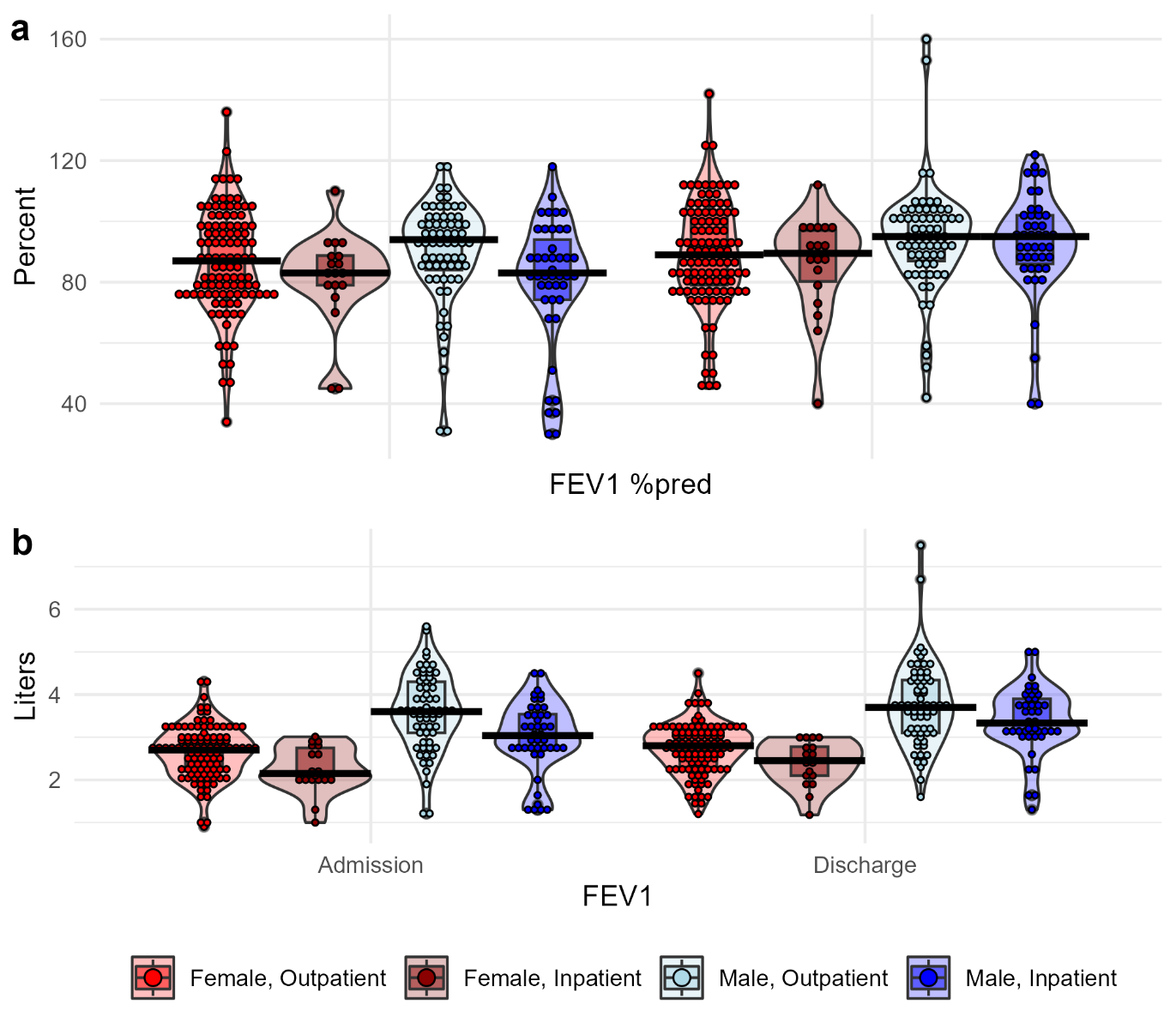


**Supplementary Figure 4.** Violin boxplot diagrams of 1-minute forced expiratory volume (FEV1). Colors indicate sample stratification by sex and SARS-CoV2 infection severity. Panel a) shows the ratio between achieved Watt and predicted values based on age- and sex-adjusted reference equations, panel b) raw values in liters.

**
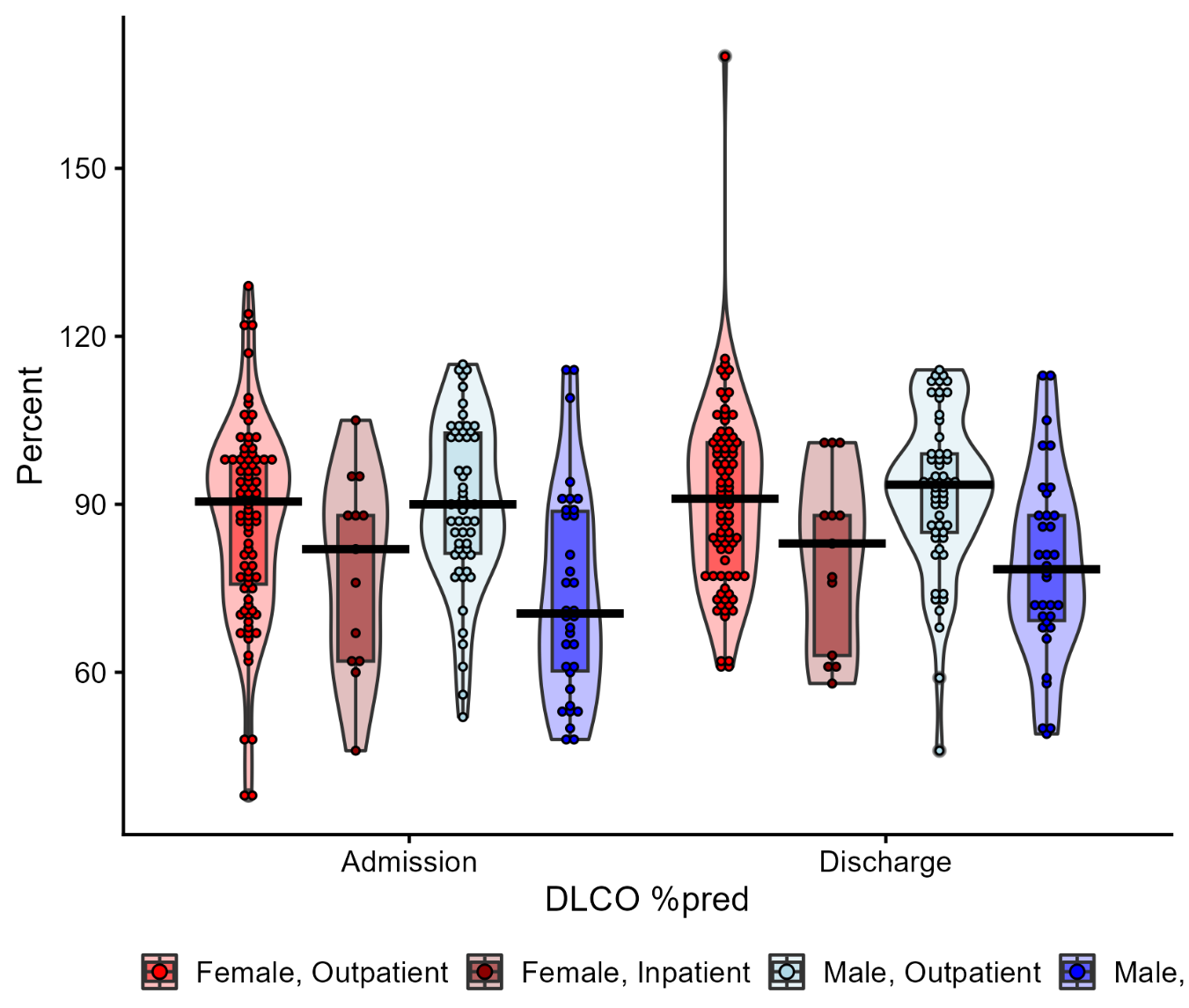
**

**Supplementary Figure 5.** Violin boxplot diagrams of the diffusion capacity of the lung (DLCO). The ratio between achieved DLCO and predicted values based on age- and sex-adjusted reference equations is depicted. Colors indicate sample stratification by sex and SARS-CoV2 infection severity.
